# Blood vessel remodeling in late stage of vascular network reconstruction is essential for peripheral nerve regeneration

**DOI:** 10.1002/btm2.10361

**Published:** 2022-06-17

**Authors:** Gang Wang, Panjian Lu, Pingping Qiao, Ping Zhang, Xiaodong Cai, Leili Tang, Tianmei Qian, Hongkui Wang

**Affiliations:** ^1^ Key Laboratory of Neuroregeneration of Jiangsu and Ministry of Education, Co‐Innovation Center of Neuroregeneration, NMPA Key Laboratory for Research and Evaluation of Tissue Engineering Technology Products Nantong University Nantong China; ^2^ Medical College of Soochow University Suzhou China

**Keywords:** blood vessel three‐dimensional reconstruction, inflammation, peripheral nerve regeneration, tissue engineered nerve, vascular remodeling, vascularization

## Abstract

One of the bottlenecks of advanced study on tissue engineering in regenerative medicine is rapid and functional vascularization. For a deeper comprehension of vascularization, the exhaustive, dynamic, and three‐dimensional depiction of perfused vascular network reconstruction during peripheral nerve regeneration was performed using Micro‐CT scanning. The 10 mm defect of sciatic nerve in rat was bridged by the autologous or tissue engineered nerve. The blood vessel anastomosis between nerve stumps and autologous nerve accomplished at 4 days to 1 week after surgery, which was a sufficient basis for the mature vascular network re‐establishment. The stronger ability for sprouting angiogenesis and vascular remodeling of autologous nerve compared with tissue engineered nerve was revealed. However, common phases of vascularization in peripheral nerve regeneration were painted: hypoxic initiation, sprouting angiogenesis, and remodeling and maturation. The effect of less‐concerned vascular remodeling on nerve regeneration was further analyzed after nerve crush injury. The blockage of vascular remodeling in late stage by VEGF injection significantly inhibited axons and myelin sheaths regeneration, which attenuated the impulse conduction toward reinnervated muscles. It was illustrated that a large amount of immature blood vessels rather than necessary vascular remodeling elevated local inflammation level in nerve regeneration microenvironment. The figures inspired us to understand the close connections between vascularization and peripheral nerve regeneration from a broader dimension to achieve better constructions, regulations and repair effects of tissue engineered nerves in clinic.

**Abbreviations**
ANOVA: one‐way analysis of variance
CT: computed tomography
CMAPs: compound muscle action potentials
DMEM: Dulbecco's modified Eagle's medium
HE: hematoxylin–eosin
SD: Sprague–Dawley
SKPs: skin‐derived precursors
SKP‐SCs: Schwann cells differentiated from skin‐derived precursors
VEGF: vascular endothelial growth factor

## 1 INTRODUCTION

Tissue engineering is the most promising technical approach for regenerative medicine realization in clinic.^1^, ^2^, ^3^, ^4^, ^5^ The purpose of researches is not only to repair the defect morphologically, but also to restore the physiological function, in which vascular network reconstruction is a premise and the basis for graft survival. So far, one of the key points and bottleneck problems is the vascularization of tissue engineered tissues and organs.^6^, ^7^, ^8^, ^9^ Blood vessels are regenerated, once the maximum free diffusion distance of oxygen about 200 μm is exceeded.^10^, ^11^, ^12^ Therefore, the rapid establishment of an effective blood circulation with the surrounding tissues after implantation is crucial for the successful regeneration.

The same is true for peripheral nerve regeneration studies. Construction strategies of tissue engineered nerves have developed from only biomaterial nerve conduits to tissue engineered nerves involving supporting cells, growth factors or cell matrices.^13^, ^14^, ^15^, ^16^, ^17^ Unfortunately, in both animal experiments and clinical trials, repair effects of tissue engineered nerves under existing strategies are difficult to fully achieve or even surpass those of autologous nerves. Autologous nerve transplantations are still the gold standard for clinical peripheral nerve defect repairs.^18^, ^19^, ^20^, ^21^ It was confirmed that migrations of Schwann cells were guided by vascular endothelial cells in peripheral nerve regeneration, which proved the close relationship between angiogenesis and nerve regeneration in morphology.^22^ Meanwhile the intimate collaboration between peripheral nerve regeneration and angiogenesis at the molecular regulation level was demonstrated.^23^ It is widely realized that vascular network reconstruction is an important part of nerve regeneration microenvironment, deficiency of which is the key constraint for advanced improvement of repair effects of tissue engineered nerves.^24^, ^25^

Few basic and systematic researches on vascularization in peripheral nerve regeneration were performed. Alternatively, most studies focused on application explorations. Supplements of proangiogenic factors or incorporations of endothelial cells and stem cells were adopted to promote the neovascularization of tissue engineered nerve.^26^, ^27^, ^28^, ^29^, ^30^, ^31^ Yet it is far from enough to only conduct application explorations for breakthrough of angiogenesis bottleneck to more effectively promote peripheral nerve regeneration. How vascular networks are reconstructed step by step in peripheral nerve regeneration? Are the more blood vessels the better peripheral nerve regeneration in different phases? What are the differences of vascularization in nerve regeneration between tissue engineered and autologous nerve grafts? Lots of important processes and key regulations are still unclear. Fundamental researches in‐depth are required to reveal the entire vascular network reconstruction during peripheral nerve regeneration including details, as well as its effects on regeneration microenvironment.

Vascularization of autologous nerve repair, as a gold standard in clinic, has important inspirations and great references. In our research, the tissue engineered nerve was constructed with Schwann cells differentiated from skin‐derived precursors (SKP‐SCs) as supporting cells and chitosan nerve conduits combined with silk fibroin fibers as scaffolds. Allogeneic cells improve the local nerve regeneration microenvironment by secreting a variety of growth factors during their survival period, thereby promoting peripheral nerve regeneration well. The sciatic nerve defect in rat was bridged by the tissue engineered nerve or the autologous nerve to observe the vascularization in peripheral nerve regeneration. Based on the detailed and objective depiction, the main stages of vascular network reconstruction were further analyzed to reveal the important influences on peripheral nerve regeneration. The results painted a panoramic picture of the similarities and differences in vascular network reconstruction by different repair methods. More importantly, it is recognized that vascularization in peripheral nerve regeneration is quite complicated, and each stage directly play an important role on nerve regeneration. Especially, the tight link between nerve regeneration and vascular remodeling was identified. This study provides a more comprehensive and in‐depth understanding of vascularization and microenvironment in peripheral nerve regeneration, which is the theoretical basis and new inspiration to precisely regulate the construction of vascularized tissue engineered nerve for better clinical peripheral nerve injury repair.

## 2 METHODS

### 2.1 Tissue engineered nerve construction

Tissue engineered nerves were constructed in vitro including chitosan nerve conduits inserted with silk fibroin fibers as scaffolds and SKP‐SCs as supporting cells. In brief, the chitin/chitosan (Nantong Xincheng Biochemical, Nantong, China) mixtures were injected into stainless‐steel casting molds, which were then sealed and placed at −12 °C for 2–4 h. Then the conduits were lyophilized under a 35–45 mTorr vacuum for 20 h after rinsing. The porous chitosan conduits were 2 mm inner diameter, 3 mm outer diameter.^32^ Skin‐derived precursors (SKPs) of newborn Sprague–Dawley (SD) rats were isolated and differentiated to SKP‐SCs, then amplificated in vitro.^33^, ^34^ The SKP‐SCs and scaffolds were co‐cultured with 37 °C and 5% CO_2_ for sufficient contact. Briefly, SKP‐SCs resuspended in Dulbecco's modified Eagle's medium (DMEM) were added to the silk fibroin fibers and chitosan nerve conduits followed initial adhesion for 4–6 h, which was performed again after the scaffolds were turned over. The artificial nerves and SKP‐SCs were co‐cultured in DMEM for 2 days and then in DMEM supplemented with 50 μg/ml ascorbic acid (Sigma) for additional 12 days. The cells homogeneously adhered to the surface of fiber and conduit. The final cell density was 10^6^/ml.^35^ Tissue engineered nerves were stored in NS following rinse twice with NS.

### 2.2 Sciatic nerve injury surgery

Adult 6–8 week old female SD rats (200–220 g) were provided by the Experimental Animal Center of Nantong University (License No. SYXK (Su) 2017‐0046). The animals were randomly divided into different groups (Table 1). The rats were housed in a temperature‐controlled environment and allowed food and water ad libitum. The Administration Committee approved all experimental protocols of Experimental Animals, following the guidelines of the Institutional Animal Care and Use Committee, Nantong University, China (Inspection No: 20180301‐009). The SD rats were deeply anesthetized with an intraperitoneal injection of a compound anesthetic.^20^ The skin and muscle were incised to expose the sciatic nerve at the left mid‐thigh. An 8 mm segment of the sciatic nerve was resected to produce a 10 mm gap after slight retraction.^36^ The nerve defect was bridged by the tissue engineered nerve (TEN) or autologous nerve with a 180° reversal of the ipsilateral sciatic nerve after dissection (Auto). Compared with the defect model, the expected good regeneration of crush model is more convenient for the nerve regeneration study. For the nerve crush model, the sciatic nerve was exposed carefully and crushed 3 mm for 30 s in the middle segments using hemostatic forceps. The 6 μl of 1 μg/ml vascular endothelial growth factor (VEGF) recombinant protein was injected perineurally at the injury site immediately after nerve injury. In the control group, to exclude possible influences of the injection operation and pressure on nerve regeneration, the 6 μl of saline was also injected perineurally.^37^ Then, the muscle layer and skin were closed with sutures. After surgery, the animals were placed in warmed cages.

**TABLE 1:** The number of animals for analysis

Injury	Sciatic nerve defect bridging (tissue engineered or autologous nerve)								Sciatic nerve crush					
									No treatment			VEGF or saline injection		
Analysis	Vascular reconstruction/stereoscopic microscope and HE								Vascular staining			Microstructure	Ultrastructure	Function
Time	1 d	4 d	1 w	2 w	3 w	4 w	8 w	12 w	7 d	21 d	28 d	28 d	28 d	28 d
Number	3/3	3/3	3/3	3/3	3/3	3/3	3/3	3/3	3	3	3	3	3	5

### 2.3 Electrophysiology detection

Under deep anesthesia, the crushed sciatic nerve was re‐exposed. Electrical stimuli were applied to the nerve trunk at distal and proximal ends of crushed segment sequentially. Compound muscle action potentials (CMAPs) were recorded on the gastrocnemius belly. The detection of normal CMAPs was performed at the uninjured contralateral side.^38^

### 2.4 Stereoscopic observation and histological assessment

The rats were deeply anesthetized with an intraperitoneal injection after surgery at different time points. The tissue engineered nerves or autologous nerves were fully dissociated and exposed. The bridge segments were placed in the vision of stereomicroscope (AZ100, Nikon). The surface blood vessels were focused and photographed. In addition, the tissue engineered nerves, autologous nerves and crushed nerves were harvested, fixed and frozen sliced into cross sections followed by hematoxylin–eosin (HE) staining. The regenerated nerves after crush were also performed to a special trichrome staining, for which three main dyes (hematoxylin, Fast Green FCF and Chromotrope2R) were used.^16^ The slices stained by HE and trichrome were observed and photographed in light microscope (AxioImager M2, Zeiss). The three random fields with high magnification (×400) of crushed nerves per animal were selected for the inflammation analysis.

### 2.5 Blood vessel three‐dimensional reconstruction

At different time points after surgery, the rats were deeply anesthetized with the anesthetic again. The animals were infused with about 500 ml NS mixed with 0.8 ml heparin sodium (Changzhou Qianhong Pharmaceutical Co., Ltd, Changzhou, China) with a final concentration of 10 U/ml by the pinhead inserted into the left ventricular. Then blue Microfil compounds (Flow Tech, Inc., Carver, MA) were infused with a 50 ml syringe through the aorta. The perfusions were not ended until the contrast agents outflow from the right atrium and the skins of foot become blue.^35^ Finally, the root of aorta was ligated with surgical sutures. The rats were placed in a refrigerator with 4 °C overnight for the curing of contrast agents. After the curing of contrast agents, the surgical sites were reopened to expose the tissue engineered nerves or autologous nerves, which were carefully dissected and transparented in a gradient glycerin to select the samples with well perfusions of micro vessels for Micro‐computed tomography (CT) scanning. The samples were scanned by SkyScan1172 Micro‐CT (Bruker Corporation, Billerica, MA) under conditions of voltage 40 kV, current 250 μA and resolution 7.96 μm. The scanning of each sample took about 22 min. The images of three‐dimensional blood vessels were reconstructed, and vascular parameters including number, size and connectivity were analyzed in a unified manner by the software SkyScan CTVOX 2.1.

### 2.6 Immunofluorescence

The sections in the middle segments of crushed nerves were blocked with 5% goat serum for 1 h at 37 °C, incubated with primary antibodies overnight at 4 °C, and then incubated with secondary antibodies for 1 h at room temperature. Primary antibodies included mouse anti‐NF200 antibody (1:200 dilution, Sigma), rabbit anti‐S100 antibody (1:200 dilution, Abcam), goat anti‐CD34 antibody (1:50 dilution, R&D), mouse anti‐α‐SMA‐FITC antibody (1:300 dilution, Sigma), mouse anti‐CD68 antibody (1:100 dilution, Abcam), and rabbit anti‐CD206 antibody (1:100 dilution, Abcam). Secondary antibodies included goat anti‐mouse IgG‐Alex‐488 (1:400 dilution, Abcam), sheep anti‐rabbit IgG‐Cy3 (1:1000 dilution, Abcam), donkey anti‐goat IgG‐Alex‐488 (1:500 dilution, Abcam), donkey anti‐goat IgG‐Alex‐647 (1:200 dilution, Abcam), donkey anti‐mouse IgG‐Alex‐488 (1:400 dilution, Abcam), and sheep anti‐rabbit IgG‐Cy3 (1:1000 dilution, Abcam). Nuclei were marked using Hoechst 33342 (1:5000 dilution, Life Technologies). Images were acquired under fluorescence microscopy (Zeiss). The three random fields with high magnification (×400) per animal were selected for the statistical analysis.

### 2.7 Transmission electron microscope

The regenerated nerves of crushed segments were collected, postfixed in 4% glutaraldehyde, and embedded in Epon 812 epoxy resin (Sigma).^19^ Ultrathin sections were obtained and stained with lead citrate and uranyl acetate. The morphology of nerves was observed under a transmission electron microscope (JEOL Ltd., Tokyo, Japan). The three random fields with low magnification (×1.2 k) per animal were used for myelinated nerve fiber statistics involving the g‐ratio (nerve axon diameter/nerve fiber diameter), average diameter of axons and average thickness of myelin. The five random fields with high magnification (×20.0 k) per animal were selected for the analysis of myelin sheath layers.

### 2.8 Statistical analysis

The data were presented as means ± SD. The sample sizes for statistical analysis were displayed in Table 1. Comparisons between two groups were carried out with Student's *t* test using Graph‐Pad Prism 6.0 software (GraphPad Software Inc., La Jolla, CA). One‐way analysis of variance (ANOVA) was used for comparisons of more than two groups by Stata 7.0 software package (Stata Corp., College Station, TX). Differences were considered significant at *p* value <.05.

## 3 RESULTS

### 3.1 Vascular network of the normal sciatic nerve

**FIGURE 1:**
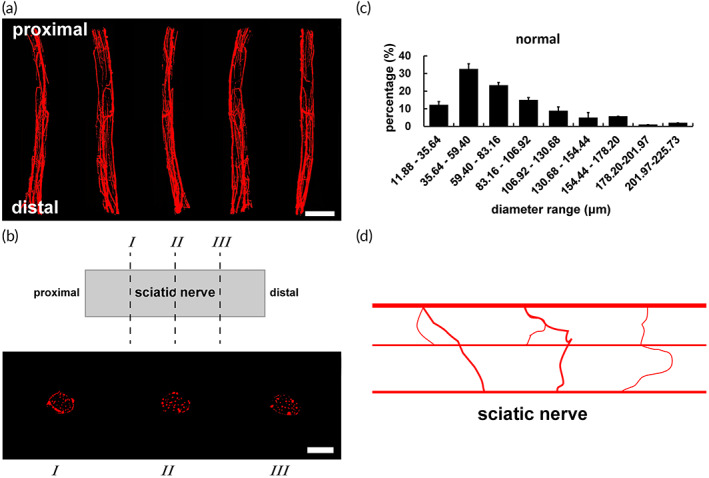
Microvascular network of the normal sciatic nerve. Micro vessels of the vascular network were demonstrated in red. (a) Longitudinal three‐dimensional reconstruction images in different angles. Scale bar, 2000 μm. (b) Images in different cross sections. *I*, *II*, and *III* instructed the quarter, half and three quarter sites from proximal to distal, respectively. Scale bar, 1000 μm. (c) Graph showed the diameter range of blood vessels (*n* = 3). (d) The diagram showed longitudinal vessels and traffic branches.

A complex vascular network of the normal sciatic nerve was formed by interconnections of abundant blood vessels, most of which were distributed along the longitudinal axis of the nerve with traffic branches to anastomose each other (Figure 1a,b). The diameters of blood vessels ranged from 11.88 to 225.73 μm, which were micro vessels and capillaries (Figure 1c). The vast majority of blood vessels in vascular network were <100 μm in diameters. Micro vessels in diameters of 11.88–106.92 μm accounted for 83.19% including 32.60% of 35.64–59.40 μm and 23.34% of 59.40–83.16 μm (Figure 1c). The sum of micro vessels in diameters of 35.64–83.16 μm reached a maximal proportion of 55.94%, which was more than half of total.

### 3.2 Micro vessels anastomosis following sprouting from two stumps of the tissue engineered nerve

**FIGURE 2:**
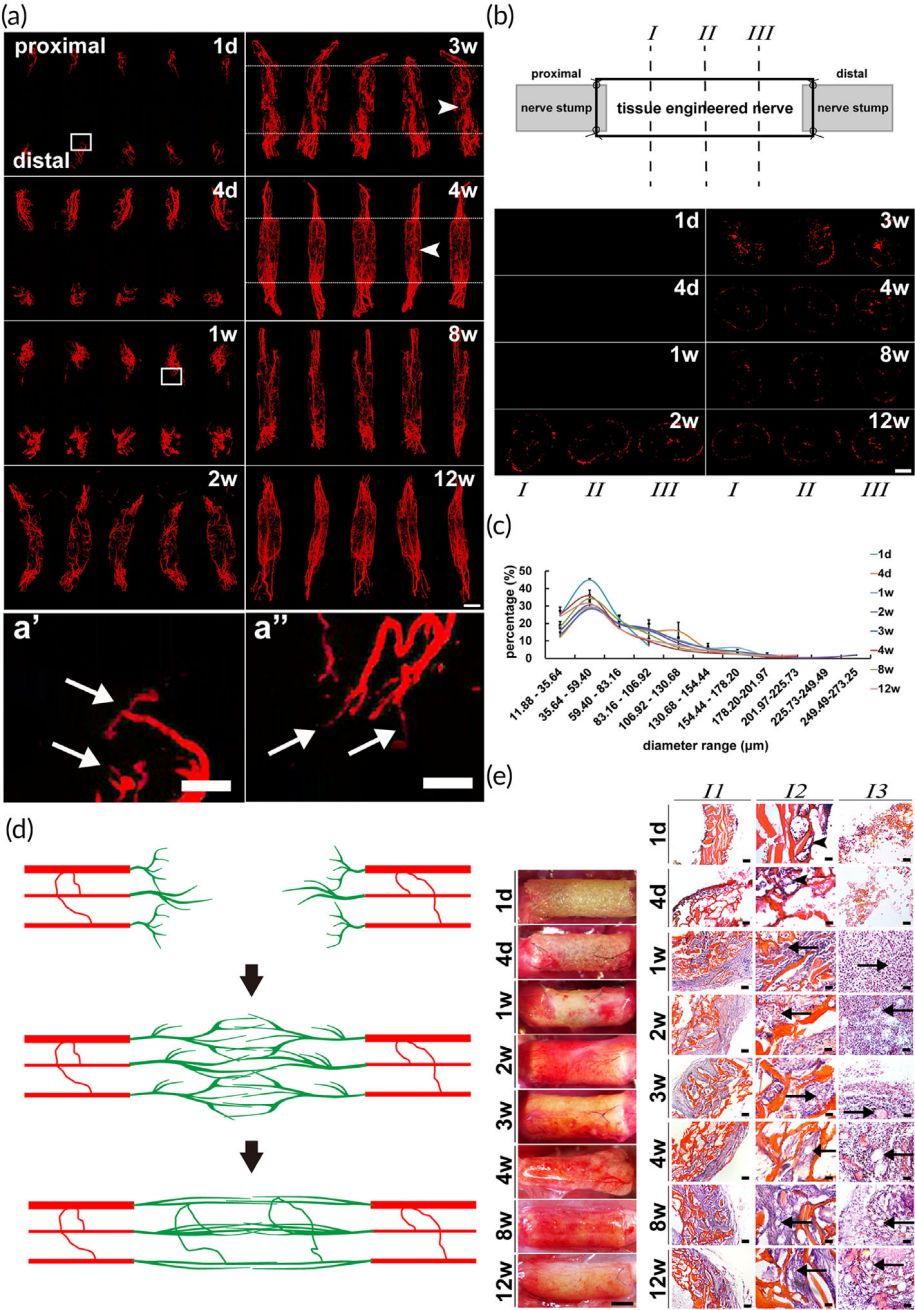
Micro vessels anastomosis in the middle segment of tissue engineered nerve. Red micro vessels of the vascular network of tissue engineered nerve were displayed. (a) Longitudinal three‐dimensional reconstruction images in different angles at each time point. The contact and anastomosis of blood vessels in the middle segment at 3 and 4 weeks after surgery were pointed by arrowheads. Scale bar, 2000 μm. (a') and (a'') Magnification of the image in white box at 1 day and 1 week, respectively, of (a). The arrow indicated frontmost branches of sprouting micro vessels. Scale bar, 500 μm in (a') and (a''). (b) Images in different cross sections at each time point. The quarter, half and three quarter sites from proximal to distal of cross sections were instructed by *I*, *II*, and *III*. Scale bar, 1000 μm. (c) Graph showed the diameter range of blood vessels at each time point (*n* = 3). (d) The diagram showed dynamic progress of vascular network reconstruction of tissue engineered nerve. Red indicated micro vessels of the nerve stumps. Green indicated the new micro vessels. (e) Representative photographs of tissue engineered nerve showed the surface blood vessels by stereoscopic observation and the inner blood vessels in the middle segment by HE staining. *I1* (low magnification) and *I2* (high magnification) showed micro vessels in the wall of tissue engineered nerve. *I3* (high magnification) showed micro vessels in the regenerated nerve tissue of tissue engineered nerve. Arrowhead indicated the vascular lumen‐like structures. Arrow indicated the micro vessels. Scale bar, 2000 μm in stereoscopic photographs. Scale bar, 100 μm in *I1*. Scale bar, 20 μm in *I2* and *I3*.

New micro vessels sprouted mainly from two directions of the nerve stumps, and also a few of blood vessels from surrounding connective tissues grew into the wall and lumen of nerve conduit. The growth of blood vessels originating from both nerve stumps was initiated at 1 day, 4 days, and 1 week followed a significant increase and extension to the middle segment of graft at 2 weeks after surgery (Figure 2a). Tiny branches in the tip of sprouting vessels were visible showing the sufficient contrast agent perfusion for objective display of microvascular network (Figure 2a' and a''). Then neovascularization sprouting from both ends of the graft contacted and anastomosed in the middle segment at 3 and 4 weeks after surgery (Figure 2a). At later stage of 8 and 12 weeks, the blood vessels in middle segment further anastomosed and reconstructed forming a bundle of distinct vascular bridge in the center of lumen (Figure 2a). Further observations of the cross sections were performed. The largest amount and also the most widespread spatial distribution of blood vessels appeared at 3 weeks after surgery (Figure 2a,b). Then most of the neovascularization were concentrated in the center of lumen with a reduced spatial distribution and fewer blood vessels near the conduit wall at 4, 8, and 12 weeks after surgery (Figure 2a,b). The basic trend of diameter distribution with a largest proportion of 35.64–59.40 μm at all‐time points was displayed (Figure 2c). The diameter distribution was relatively close to that of the normal nerve at each time point except at 1 day after surgery (Figures 1c and 2c). Meanwhile stereoscopic and HE photographs, from another aspect, illustrated the dynamic process of vascular network reconstruction in peripheral nerve regeneration. The significant increase of blood vessels was also demonstrated at the middle stage of vascularization (Figure 2e).

### 3.3 Sprouting angiogenesis based on vessel reuse of the autologous nerve

**FIGURE 3:**
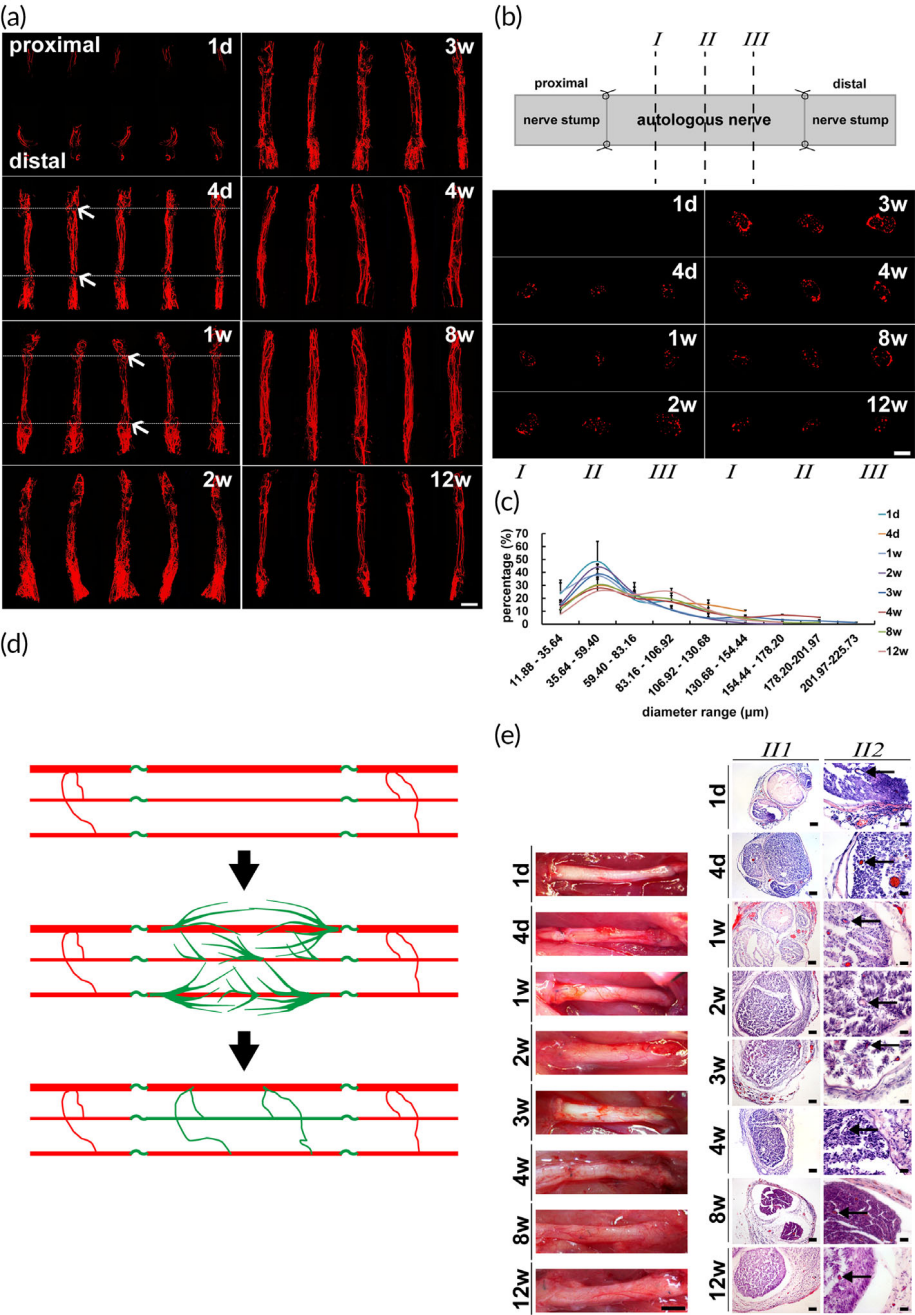
Reuse of original micro vessels in the graft segment of autologous nerve. Red micro vessels of the vascular network of autologous nerve were displayed. (a) Longitudinal three‐dimensional reconstruction images in different angles at each time point. The anastomosis and recanalization of blood vessels between the graft segment and nerve stumps at 4 days and 1 week after surgery were pointed by arrows. Scale bar, 2000 μm. (b) Images in different cross sections at each time point. The sites of cross sections at quarter, half and three quarter from proximal to distal were instructed by *I*, *II*, and *III*. Scale bar, 1000 μm. (c) Graph showed the diameter range of blood vessels at each time point (*n* = 3). (d) The diagram showed the dynamic progress of vascular network reconstruction of autologous nerve. Red indicated micro vessels of the nerve stumps and original micro vessels in the graft segment. Green indicated the new micro vessels. (e) Representative photographs of autologous nerve showed the surface blood vessels by stereoscopic observation and the inner blood vessels in the middle segment by HE staining. *II1* (low magnification) and *II2* (high magnification) showed micro vessels of the autologous nerve. Arrow indicated the micro vessels. Scale bar, 2000 μm in stereoscopic photographs. Scale bar, 100 μm in *II1*. Scale bar, 20 μm in *II2*.

A small amount of sprouting growth of blood vessels was observed from both sides of nerve stumps at 1 day after the nerve defect bridged by an autologous nerve (Figure 3a). Immediately at 4 days to 1 week after surgery, the blood vessels of bilateral nerve endings had anastomosed with those originally in bridge segment that were longitudinally parallel blood vessels rather than new sprouting micro vessels to establish an effective circulation quickly (Figure 3a). A large number of neovascularization sprouted on the basis of reused blood vessels in the bridge segment at 2 and 3 weeks reversed to a vascular network that was close to the normal in morphology at 12 weeks after surgery (Figure 3a). The largest number of blood vessels and also the largest cross‐sectional areas of graft at 2 and 3 weeks after surgery was demonstrated (Figure 3a,b). Then the number of micro vessels and cross‐sectional areas of graft reduced gradually until the reconstructed vascular network was close to morphology of normal nerve at later stage of 4, 8, and 12 weeks (Figure 3a,b). The reconstructed vascular network with a largest proportion of 35.64–59.40 μm diameters was similar to normal nerve at each time point (Figure 3c). More micro vessels in diameters of 35.64–59.40 μm were indicated at 1 day and 2 week after surgery. But the diameter distribution at 4 days was very similar to the normal nerve unlike that at other earlier stages, which revealed the micro vessels recanalization too (Figures 1c and 3c). The diameter distribution at 4, 8, and 12 weeks of later stages was closer to the normal nerve than that at 1 day, 1 week, 2 weeks and 3 weeks (Figures 1c and 3c).The surface and inner blood vessels of the autologous nerve in vascularization were also displayed by stereoscope and HE staining. The reperfusion of blood vessel in graft segment was demonstrated at 4 days and 1 week of the autologous nerve (Figure 3e).

### 3.4 Consistent progress but different efficiency of vascular network reconstruction of two repair methods

**FIGURE 4:**
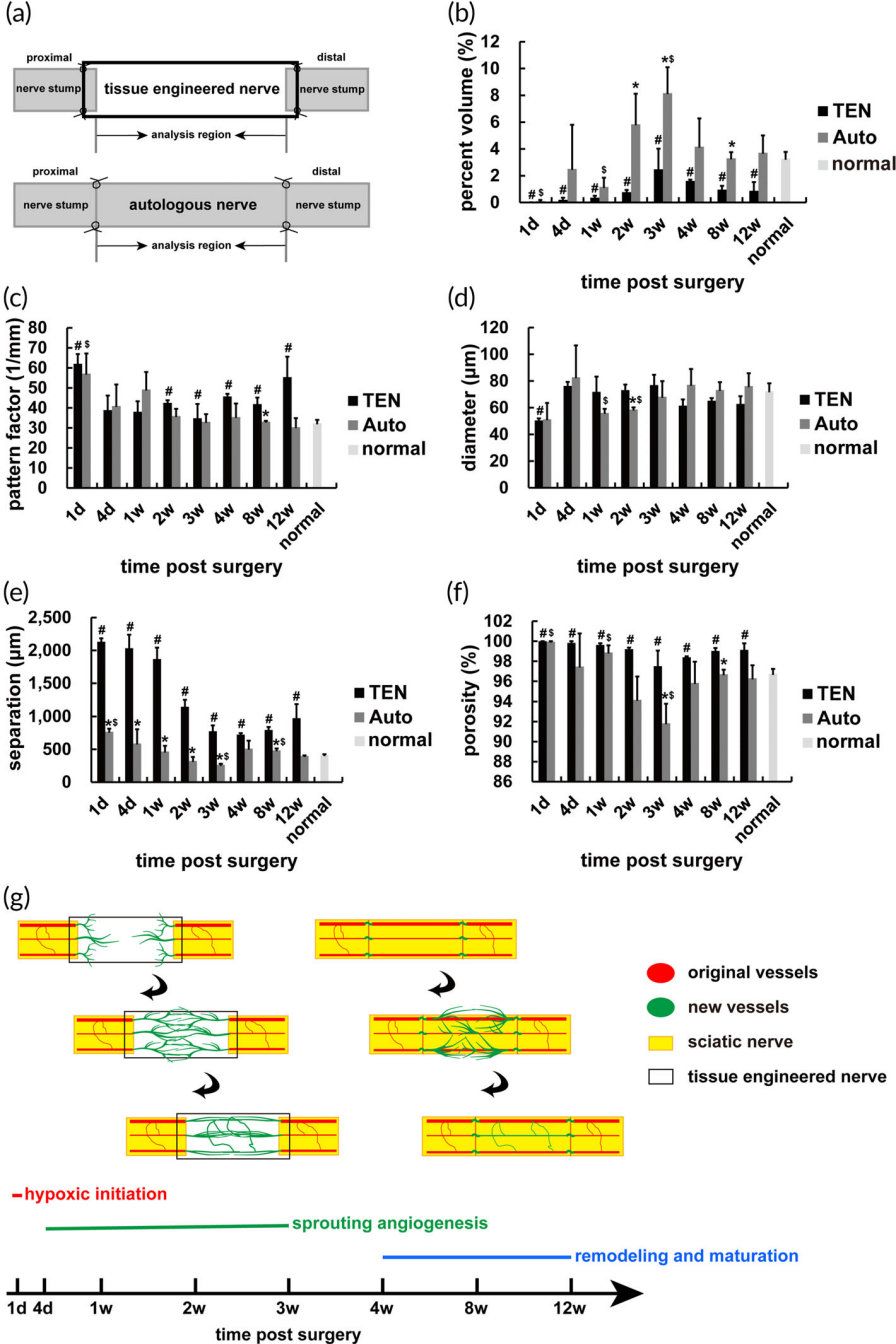
General vascular network reconstruction process with different patterns during peripheral nerve regeneration. Graph showed the parameter analysis of blood vessel three‐dimensional reconstruction images (*n* = 3). (a) The analysis region of tissue engineered nerve and autologous nerve. (b) The demonstration of statistical analysis of percent volume (%). (c) The demonstration of statistical analysis of pattern factor (1 mm). (d) The demonstration of statistical analysis of diameter (μm). (e) The demonstration of statistical analysis of separation (μm). (f) The demonstration of statistical analysis of porosity (%). *p < 0.05, TEN versus Auto at the same time point. ^#^ *p* < 0.05, TEN versus normal. ^$^ *p* < 0.05, Auto versus normal. Diagram showed the dynamic progress of vascular network reconstruction of tissue engineered nerve and autologous nerve during peripheral nerve regeneration. (g) Three stages of the biological process of vascular network reconstruction in both groups were displayed: hypoxic initiation, sprouting angiogenesis, and remodeling and maturation. The vascular bridge was formed by the anastomosis of blood vessels sprouting from nerve stumps of tissue engineered nerve. The vascular network was reconstructed on the basis of reuse of blood vessels in graft segment of autologous nerve.

Vascular related parameters of the reconstructed network of bridge segment (Figure 4a) were statistically analyzed reflecting the quantity, diameter, connectivity, and spatial distribution. The vascular volume density was applied to evaluate the amount of micro vessels. The microvascular density of autologous nerve was significantly higher than that of tissue engineered nerve at the major stages of sprouting angiogenesis (2 and 3 weeks after surgery), revealing the stronger blood vessel sprouting ability (Figure 4b). The number of blood vessels of both groups decreased at 4, 8, and 12 weeks after surgery (Figure 4b). Furthermore, at each time point, the density of micro vessels of tissue engineered nerve was significantly less than that of normal nerve (Figure 4b). The smaller the pattern factor, the better the connectivity of blood vessels. From 2 to 12 weeks after surgery, the blood vessel connectivity of tissue engineered nerve was worse than normal nerve; however, no statistical differences between the autologous nerve and normal nerve were demonstrated (Figure 4c). At 4, 8, and 12 weeks after surgery, a gradually increasing trend of vascular diameter in both groups was displayed with no significant differences to normal nerve; meanwhile the diameter of tissue engineered nerve was slightly smaller than that of autologous nerve (Figure 4d). The blood vessel separation and porosity of tissue engineered nerve reflecting the spatial distribution were significantly larger than normal nerve, and the parameters of autologous nerve were closer to normal (Figure 4e,f).

In terms of the quantity, connectivity, and spatial distribution except for diameter, the tissue engineered nerve was significantly different from normal nerve, while the autologous nerve was close to normal. Despite the numerous differences in detail, it was a common process including a stage with blood vessel increase followed by a stage with blood vessel decrease. Three phases of vascular network reconstruction were summarized: hypoxic initiation, sprouting angiogenesis, and remodeling and maturation (Figure 4g). Particularly, blood vessel remodeling was a late stage of vascularization in peripheral nerve regeneration, which co‐existed in two repair methods.

### 3.5 Inhibition of peripheral nerve regeneration following vascular remodeling blockage

**FIGURE 5:**
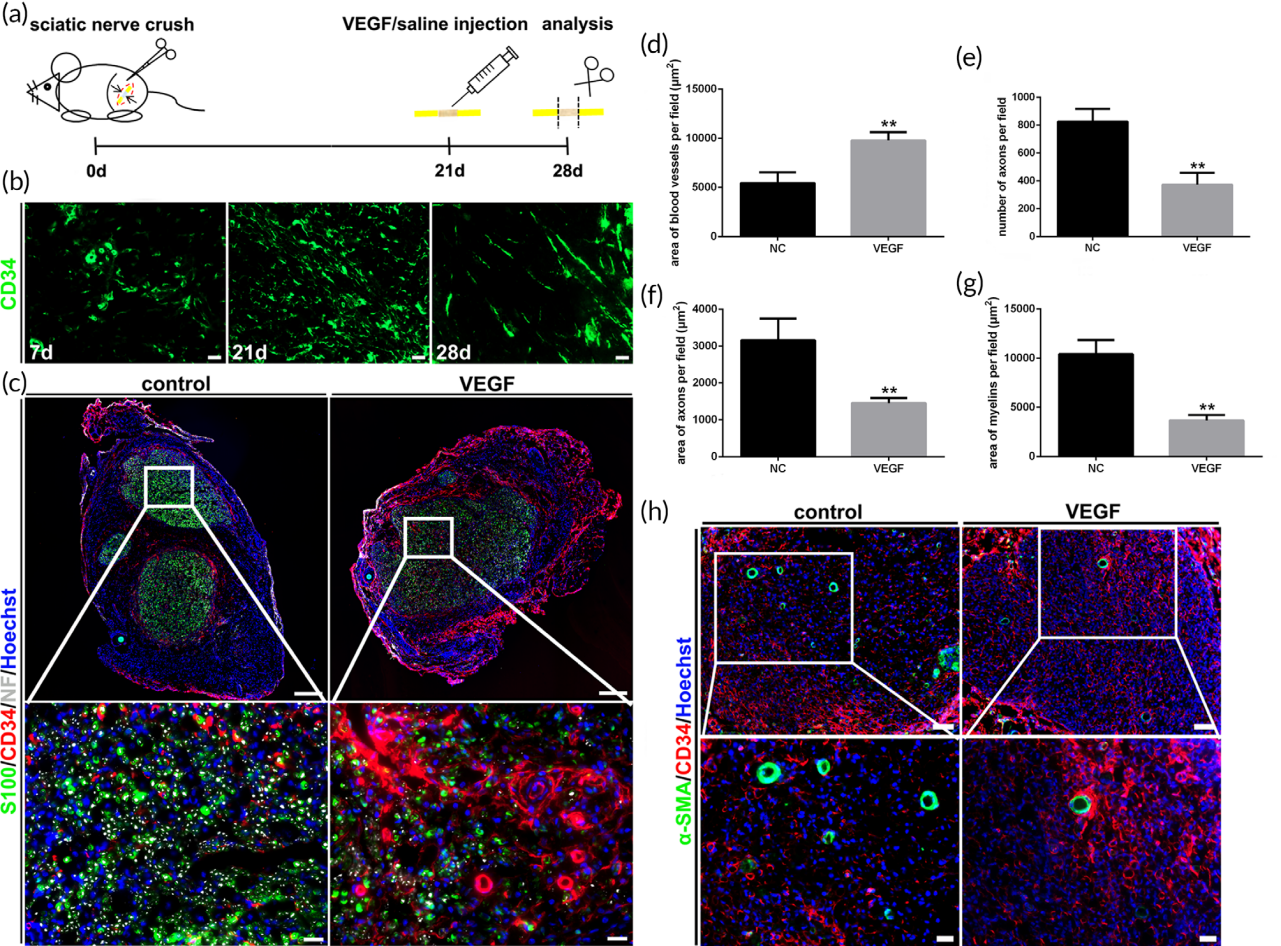
Attenuation of peripheral nerve regeneration caused by the loss of vascular remodeling. (a) Flow diagram of sciatic nerve crush and factor injection. (b) Blood vessel changes after the sciatic nerve crush injury without injection treatment. Blood vessels were green (CD34+). Scale bar, 20 μm. (c) Immunofluorescence staining of the regenerated nerves with VEGF or saline injection. A large number of new blood vessels continued to sprout due to VEGF injection, which led to significant reduction in peripheral nerve regeneration. Blood vessels were red (CD34+). Axons were white (NF+). myelins were green (S100+). Nuclei were stained using Hoechst (blue). Scale bar, 200 and 20 μm, respectively. (d–g) Histograms of the area of blood vessels, number of axons, area of axons and area of myelins (*n* = 3). There were significant statistical differences between the control and VEGF groups. **p < 0.01. (h) Immunofluorescence staining of the smooth muscle of blood vessels. The number of blood vessels surrounding smooth muscles in control group was more than that in VEGF group. Blood vessels were red (CD34+). smooth muscles were green (α‐SMA+). Nuclei were stained using Hoechst (blue). Scale bar, 50 and 20 μm, respectively.

**FIGURE 6:**
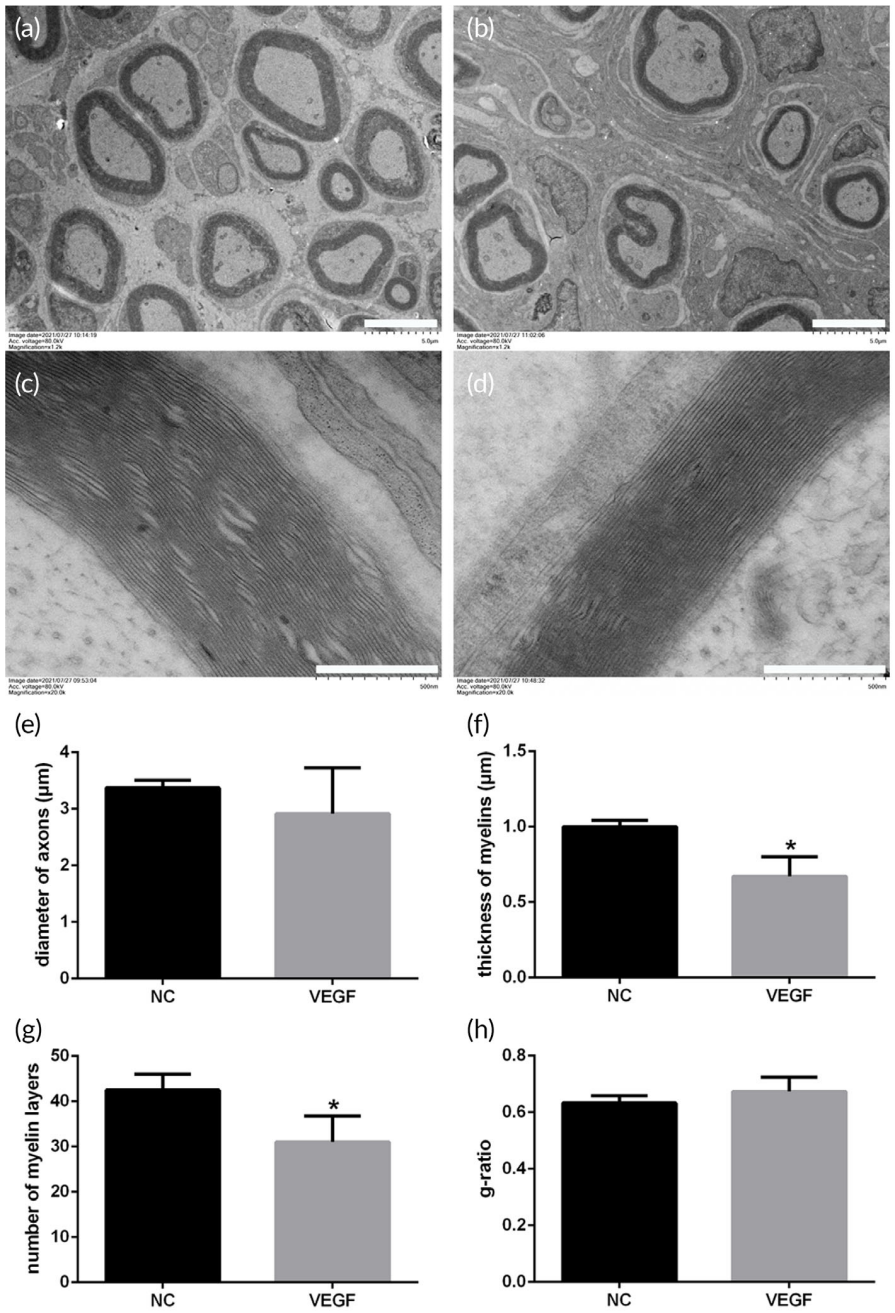
Ultrastructure of the regenerated nerve by transmission electron microscope. (a,b) Low magnified fields of the regenerated nerves. Scale bar, 5 μm. (c,d) High magnified fields of the regenerated nerves displaying myelin sheaths. Scale bar, 500 nm. (e–h) Histograms of the nerve regeneration parameters (*n* = 3). The axon diameter, myelin thickness and number of myelin layers in the VEGF group were smaller than those in the control group, and the differences of myelin thickness and number of myelin layers were statistically significant. *p < 0.05

Is the vascular remodeling in late stage of vascularization during peripheral nerve regeneration only a natural and insignificant continuation of sprouting angiogenesis, or is it also directly related to nerve regeneration? The influence of peripheral nerve regeneration after vascular remodeling blockage was further observed and analyzed (Figure 5a). A remodeling process with reduced blood vessels after nerve crush injury was also displayed (Figure 5b). The number of blood vessels was continuously maintained at a high level after VEGF injection, which was significantly different from that of the saline injection (Figures 5c,d and S1). Meanwhile, it was noted that a large number of blood vessels that disrupted the vascular remodeling were mostly small or immature vessels without surrounding smooth muscle cells (Figure 5h). On the contrary, the blood vessels in late stage of nerve regeneration in the control group showed greater maturity (Figure 5h). Then the nerve regeneration with vascular remodeling blockage was compared and analyzed. Regarding the number and size of regenerated axons and myelin sheaths, the loss of vascular remodeling severely inhibits nerve regeneration (Figure 5c and S1). It was calculated that the number of axons, areas of axons and myelins subjected to VEGF treatment were significantly less than those in the control group (Figure 5c, 5e–g). The ultrastructure of regenerated nerve further illustrated the close connection between vascular remodeling and nerve regeneration. The average myelin thickness and number of myelin layers were obviously reduced caused by the loss of vascular remodeling (Figure 6a–d, f,g). In addition, the inhibition of vascular remodeling directly affected the average diameter of regenerated axons, although the difference between two groups was not statistically significant (Figure 6a–d, e). The difference of g‐ratio was also revealed, which had no statistical significance due to the simultaneous decrease in axon diameter and myelin thickness (Figure 6h). Correspondingly, the effect of vascular remodeling disruption on nerve regeneration was also reflected in the target muscle reinnervation. The significant differences in CMAPs of gastrocnemius between the two groups were detected (Figure S2).

The representative photographs of neural trichrome staining were displayed, and the fields inside the rectangle were magnified. Due to the local injection of pro‐angiogenic factors, the number of new blood vessels in the VEGF group was significantly higher than that in the control group. Compared to the control group, the regenerated nerve fibers were significantly reduced in both number and size in the VEGF group. Myelins appear red. Axons and connective tissues appear green. Nuclei appear purple‐blue. Arrows indicated the blood vessels. Arrowhead indicated the myelinated nerve fibers. Scale bar, 50 and 20 μm, respectively.

### 3.6 Higher inflammation of regenerative microenvironment due to continuous sprouting angiogenesis

**FIGURE 7:**
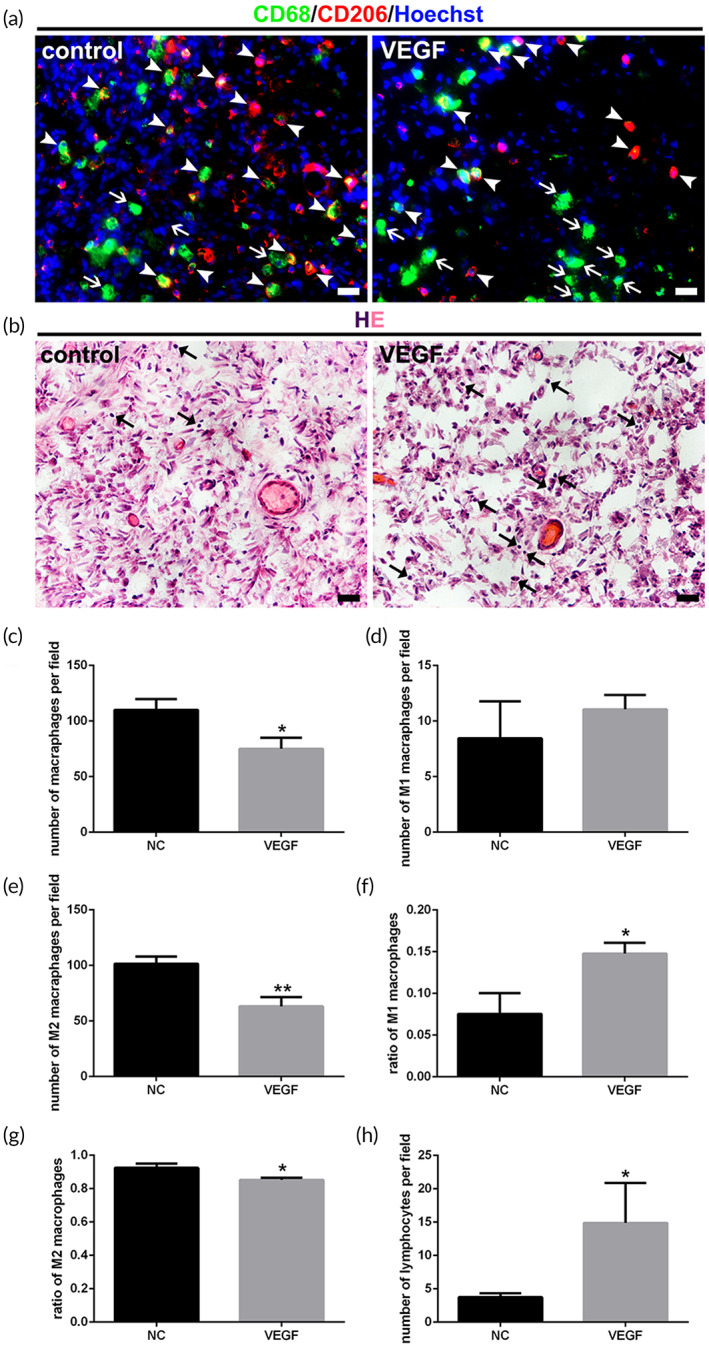
Infiltration of inflammatory cells in the peripheral nerve regeneration microenvironment. (a) Immunofluorescence staining of two subtypes of macrophages. M1 macrophages were green (CD68+). M2 macrophages were co‐localization of green and red (CD68+ and CD206+). Less M2 macrophages and more M1 macrophages were seen in the VEGF group compared with those in the control group. Arrows indicated M1 macrophages. Arrowhead indicated M2 macrophages. Scale bar, 20 μm. (b) HE staining of the regenerated nerves in control and VEGF groups. More lymphocyte infiltration was shown in the VEGF group compared with that in the control group. Arrows indicated lymphocytes with a large karyoplasmic ratio. Scale bar, 20 μm. (c–h) Histograms of the number of macrophages and lymphocytes, and the ratios of two macrophage subsets (*n* = 3). There were significant statistical differences, except for the number of M1 macrophages, between the control and VEGF groups. *p < 0.05; **p < 0.01.

The level of inflammation in peripheral nerve regeneration microenvironment is critical to nerve regeneration outcome. The number of main inflammatory cells of the regenerated nerves in crushed segments was counted and analyzed. First of all, the total number of macrophages displayed significant differences between two groups (Figure 7a,c). More M1 macrophages existed in the VEGF group, although the difference from that in the control group was not statistically significant (Figure 7a,d). Whereas, the significant decrease in the number of M2 macrophages in the VEGF group resulted in more total macrophages in the control group (Figure 7a,e). Hereafter, the in‐depth analysis of two subtypes of macrophages corresponding to the inflammatory or regenerative environment was conducted.^39^, ^40^, ^41^ The results indicated that the loss of vascular remodeling significantly increased the ratio of M1 macrophages, while the ratio of M2 macrophages was significantly lower than that of the control group (Figure 7a,d,e). The massive sprouting blood vessels in vascular remodeling stage that was contrary to normal led to a significant increase of local inflammation level. Apart from macrophages, the count of lymphocyte revealed as well that blockade of vascular remodeling raised inflammation grade in local regenerating microenvironment (Figure 7b,f).

## 4 DISCUSSION

The entire progress including details of vascular network reconstruction during peripheral nerve regeneration is quite unclear, which restricts the further improvement of tissue engineered peripheral nerve. This is the reason that prompted us to carry out such comprehensive and systematic basic research. It is believed that nerve regeneration and vascularization are closely linked.^23^, ^38^ Although the addition of more factors may increase the threshold of clinical applications, tissue engineered nerves incorporating allogeneic SKP‐SCs were designed to paint the vascularization process due to the potential to better repair peripheral nerve defects.^42^ The blood vessel three‐dimensional reconstruction by Microfil perfusion and Micro‐CT scanning was selected, which was widely used in vascular research.^43^, ^44^, ^45^ The advantage of Microfil contrast agent lies in its good filling of blood vessels and its small shrinkage after curing.^35^, ^46^, ^47^ The excellent ability for a display of micro vessels ensured an objective painting of details of vascular network reconstruction. The dynamic, meticulous and three‐dimensional illustration of vascularization in peripheral nerve regeneration was conducted. In particular, the developmental reconstruction of functional blood perfusion during nerve regeneration was revealed by the Microfil perfusion.

It was reported that increased angiogenesis leads to improved nerve regeneration to some extent in a few studies.^48^, ^49^, ^50^ Based on the description of vascular network of normal nerve, the process of vascularization in peripheral nerve regeneration was analyzed and compared between the tissue engineered nerve and autologous nerve. The different patterns of vascular network reconstruction in two repair approaches were revealed in details. The anastomosis of blood vessels and the recanalization of blood flow of autologous nerve were fairly rapid at 4 days to 1 week after surgery, which was the guidance for Schwann cell migration and axon extension.^22^ Meanwhile it was interesting that the blood vessel volume of autologous nerve at the time of vascular anastomosis at 4 days was larger than that of the normal nerve, suggesting that there may be a stage of vascular cavity expansion, whose role and significance need further research. Moreover, blood vessels of autologous nerve at 1 week were less than normal revealing that maybe just the part of original blood vessels anastomosed to be the basis of sprouting angiogenesis, which is another unknown and complex question. Compared with the tissue engineered nerve, the reuse of original blood vessels of autologous nerve aroused more rapid, efficient and mature vascular network reconstruction. It is likely that the differences in vascular network reconstruction are one of key factors affecting repair effects of tissue engineered nerve. Although the vascularization patterns of two methods are dissimilar, it is a common progress that a sprouting stage and a remodeling stage. This similarity allows us to understand and grasp the connection between angiogenesis and nerve regeneration from a higher perspective and a broader dimension. Then the coexistence of differences and similarities makes us to view and solve problems dialectically, without losing sight of bias too.

Among three phases of vascularization during peripheral nerve regeneration, vascular remodeling is the stage that we are more interested in. Is it just a natural continuation and ending of the previous stage of vascularization? A preliminary investigation on the influence of vascular remodeling on nerve regeneration was performed. The crush injury replaced the defect bridging because of the simple operation, short experimental period, and more importantly, a good regeneration process. It was revealed definitely that vascular remodeling was not a dispensable role. The loss of vascular remodeling had an impact on regeneration of both axons and myelin, especially hindering increase of myelin thickness. The further analysis of macrophage and lymphocyte infiltration confirmed that the level of inflammation in regenerative microenvironment was significantly up‐regulated due to the blockade of vascular remodeling. The loss of vascular remodeling exerted more influence on M2 macrophages; but it seemed that it caused a large number of M2 macrophages to emigrate rather than just an impact on macrophage polarization, which attracted us and needed further exploration. Whether the vascular remodeling directly or indirectly affects peripheral nerve regeneration through other chemical or physical factors remain to be studied in depth. In addition, behind phenomenon, research of deep‐level molecular regulation mechanism will provide a theoretical basis for better understanding of local peripheral regenerative microenvironment, regulation of vascularization, and relationship between vascular remodeling and peripheral nerve regeneration. It is worth investing considerable efforts in conducting related basic research, although it is more difficult. Apart from the above shortcomings, given the differences of individual animals and the complexity of environment in vivo, the sample size was relatively small. Moreover, the quantitative analysis based on three‐dimensional constructions of blood vessels, with the continuous improvement of resolution, etc., the data will be more accurate.

This study brings us two enlightenments and possible directions to improve tissue engineered nerve from the vascularization perspective: prevascularization of tissue engineered nerve, and promotion of anastomosis and recanalization of blood vessels; combined regulation of early acceleration of sprouting angiogenesis, and later improvement of vascular remodeling and maturity. Our findings lay a solid foundation for advanced study of vascularization in peripheral nerve regeneration and better nerve regeneration of tissue engineered nerves.

## AUTHOR CONTRIBUTIONS

**Gang Wang:** Data curation (equal); formal analysis (lead); investigation (equal); methodology (supporting); visualization (equal). **Panjian Lu:** Data curation (equal); formal analysis (supporting); investigation (equal); visualization (equal). **Pingping Qiao:** Investigation (supporting); validation (lead). **Ping Zhang:** Investigation (supporting); methodology (supporting). **Xiaodong Cai:** Investigation (supporting); methodology (supporting). **Leili Tang:** Validation (supporting). **Hongkui Wang:** Conceptualization (lead); methodology (lead); project administration (lead); supervision (lead); visualization (equal); writing – original draft (lead); writing – review and editing (lead). **Tianmei Qian:** supervision; writing – original draft.

## FUNDING INFORMATION

This study was supported by grants from the National Natural Science Foundation of China (Grant No. 81901256, 81873767), Jiangsu Provincial Key Medical Center and Priority Academic Program Development of Jiangsu Higher Education Institutions (PAPD).

## CONFLICT OF INTEREST

The authors declare no conflict of interest.

### PEER REVIEW

The peer review history for this article is available at https://publons.com/publon/10.1002/btm2.10361.

## ETHICS STATEMENT

Animal procedures were approved by the Administration Committee of Experimental Animals, Jiangsu Province, China and conducted in accordance with Institutional Animal Care Guidelines of Nantong University, Nantong, China (Inspection No: 20180301‐009).

## Supporting information


**FIGURE S1** Trichrome staining of the regenerated nerve

**FIGURE S2** Electromyography of the reinnervated muscles. (a) The electrophysiological waveforms of gastrocnemius under the 10 mV stimulation condition. The Stim 1 and Stim 2 channels were the CMAP records of proximal and distal ends of crushed nerves, respectively. (b) Histograms of the CMAPs of gastrocnemius at the proximal ends of crushed segments (*n* = 5). The CMAPs after VEGF injection were significantly lower than those of the control group. *p < 0.05.

## Data Availability

All data generated or analyzed during this study are included in this published article and its supplementary information files.
