# Supplementary figures and images for: Blood vessel remodeling in late stage of vascular network reconstruction is essential for peripheral nerve regeneration
Source: Bioeng Transl Med. 2022 Jun 17;7(3):e10361. doi: 10.1002/btm2.10361 (PMC9472024; doi:10.1002/btm2.10361)

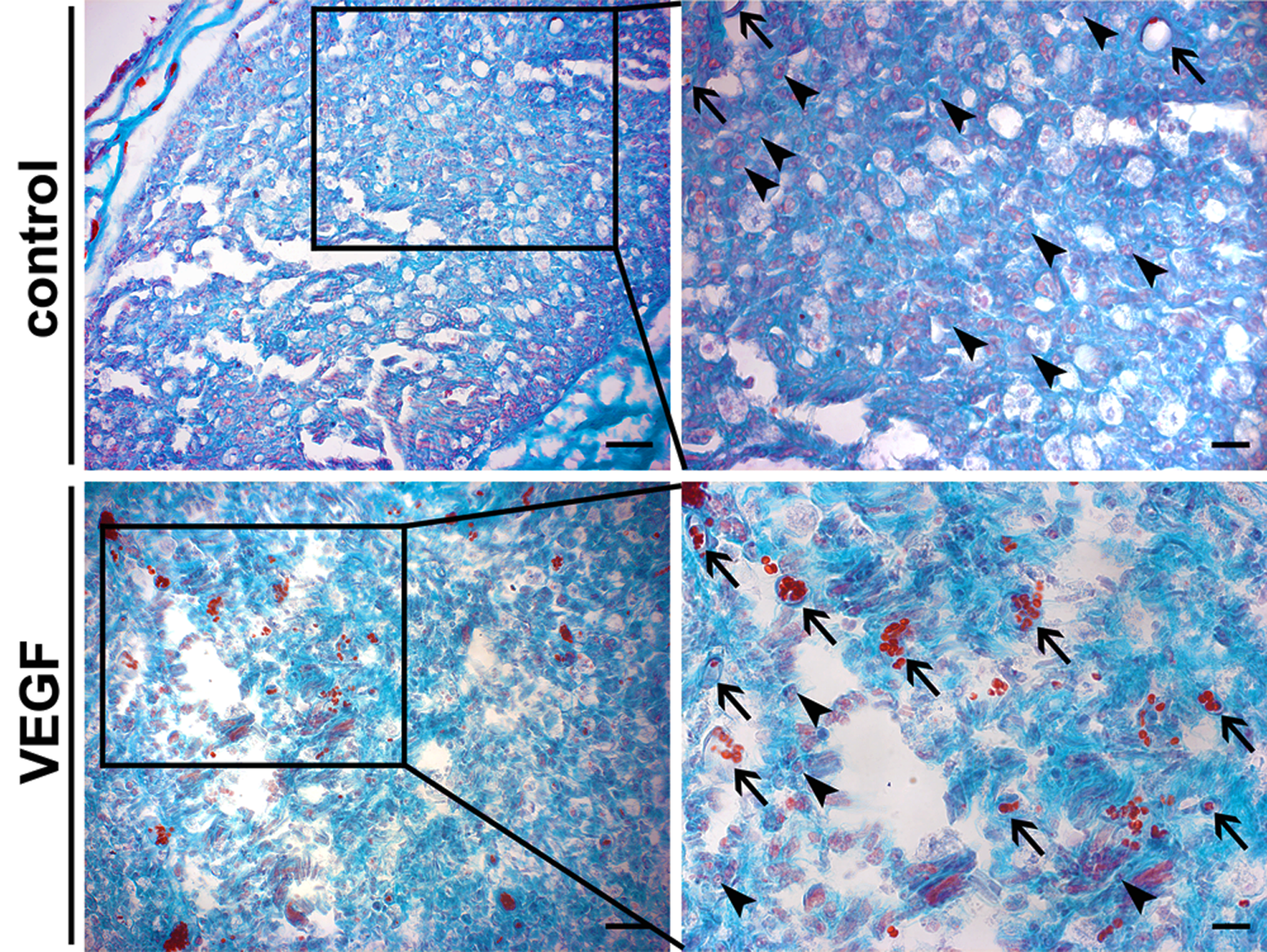

Supplement: Supplementary file 1 — FIGURE S1 Trichrome staining of the regenerated nerve [file BTM2-7-e10361-s002.tif]

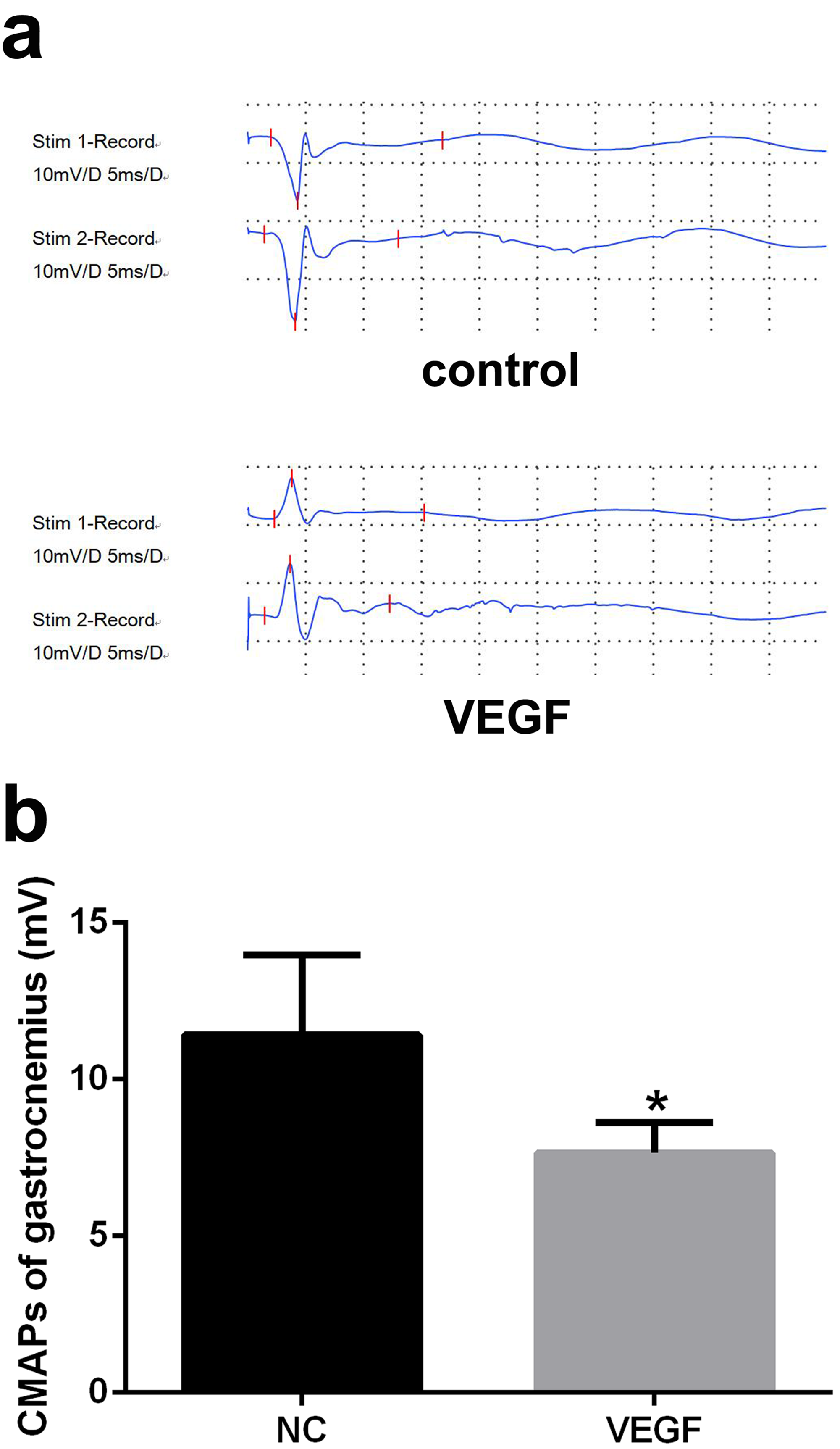

Supplement: Supplementary file 2 — FIGURE S2 Electromyography of the reinnervated muscles. (a) The electrophysiological waveforms of gastrocnemius under the 10 mV stimulation condition. The Stim 1 and Stim 2 channels were the CMAP records of proximal and distal ends of crushed nerves, respectively. (b) Histograms of the CMAPs of gastrocnemius at the proximal ends of crushed segments (n = 5). The CMAPs after VEGF injection were significantly lower than those of the control group. *p < 0.05. [file BTM2-7-e10361-s001.tif]
